# Validity Evidences of the Prefrontal Symptoms Inventory for the Elderly Brazilian Population

**DOI:** 10.6061/clinics/2020/e1863

**Published:** 2020-11-26

**Authors:** Olívia Dayse Leite Ferreira, Leopoldo Nelson Fernandes Barbosa, João Carlos Alchieri

**Affiliations:** I Programa de Pos-Graduacao em Ciencias da Saude, Campus Universitario Natal, Universidade Federal do Rio Grande do Norte (UFRN), RN, BR.; II Departamento de Psicologia, Faculdade Pernambucana de Saude (FPS), Recife, PE, BR.

**Keywords:** Alzheimer's Disease, Brazilian, Elderly, Prefrontal Symptoms, Psychometric Properties

## Abstract

**OBJECTIVES:**

This study aimed to translate the Prefrontal Symptoms Inventory (PSI) (abbreviated version) for the elderly into Brazilian Portuguese, evaluate its psychometric properties, and investigate if the PSI could distinguish between groups with (clinical group) and without (non-clinical group) a diagnosis of probable Alzheimer’s disease (AD).

**METHODS:**

The PSI was idiomatically and culturally adapted, and then administered to 256 individuals over 60 years of age who also completed a clinical interview, the Mini-Mental State Examination (MMSE), the Geriatric Depression Scale (GDS)-15, and the Frontal Assessment Battery (FAB).

**RESULTS:**

The results indicated satisfactory adjustment and adequate reliability (Ω of 0.83 and α=0.80) for the uni-factorial model. The non-clinical group showed significant correlations between the PSI-16, GDS-15, MMSE, and FAB and its six subtests. In the clinical group, there were negative correlations between the PSI-16, MMSE, and the FAB and the conceptual subtest. The groups differed statistically significantly, with the clinical sample showing the highest PSI-16 score. In the non-clinical group, there were significant positive correlations between age and PSI-16, and negative correlations between education and PSI-16.

**CONCLUSION:**

The results of this study indicate that the PSI-16 can be used as a valid and reliable screening tool for clinical use in the elderly with and without pathology.

## INTRODUCTION

The prefrontal cortex (PFC) refers to regions of the cerebral cortex anterior to the premotor cortex and supplemental motor area. It is understood to be an association area interconnected with a multiplicity of cortical and subcortical regions, which allows functional orchestration through mechanisms of control, organization, and coordination. It is postulated that this structure is related to cognition, emotion, and social behavior and these functions are controlled by independent as well as interconnected areas (1).

The PFC appears to be the brain area most affected by aging. This, and the age-related decrease in performance observed in neuropsychological tests underpinned the theory of Frontal Lobe Aging (2). The frontal lobe’s vulnerability to aging is explained by it being the last to develop in both phylogenetic and ontogenetic terms (3).

The PFC circuits are sensitive to different brain dysfunctions that cause damage to cognitive, emotional, and motivational spheres (4). From this perspective, Alzheimer’s disease (AD) is a condition in which dysexecutive symptoms are present from the onset of dementia. Cognitive changes in patients with AD have been associated with structural changes in regions such as the hippocampus, and prefrontal, parietal, and temporal cortexes on brain imaging (5).

AD symptoms involve not only cognitive, but also behavioral and affective changes, and diagnosis requires complex investigation, including neuropsychological assessment for screening and investigation purposes. Neuropsychological instruments used in cognitive screening to measure frontal changes include the Frontal Assessment Battery (FAB) (6) and the Frontal Systems Behavior Scale, which has subscales assessing apathy, disinhibition, and executive dysfunction (7).

The neuropsychological tests currently used in evaluations focus on isolated aspects only, rather than on the cooperation and integration of various cognitive, emotional, and motivational processes intrinsic to the activities of daily living (8). Additionally, they are conducted in artificial testing environments not involving everyday situations, with detracting and unpredictable stimuli that demand multiple processes.

In recent years, it has been recommended that a questionnaire or symptom inventory that investigates functioning in daily life should be used along with neuropsychological tests for measuring cognitive performance. The use of self-reported inventories enables a quantitative and qualitative understanding of the symptoms, providing wider insights than can be obtained via a clinical interview, as it enables systematic collection of information via a list of questions directed to typical situations. This type of instrument also facilitates evaluation of emotional/motivational behaviors, going beyond only inferences in the cognitive domains (9).

In this respect, the Prefrontal Symptoms Inventory (PSI), developed by Ruiz-Sánchez de León et al. (10), may be useful, as it is used *a priori* to evaluate the presence of prefrontal symptomatology in patients with addictive behavior. The PSI has demonstrated adequate psychometric properties in patients with addictive behavior (10) and those with acquired brain damage and degenerative dementia (11) in the Spanish population.

To verify the ecological and convergent validity of the PSI with neuropsychological tests (WAIS-IV, Rey’s complex figure, Stroop), Pedrero-Pérez et al. (12) studied the performance of 52 people with addictive behavior. They found that the scale of difficulty in execution of tasks is related to the ability to solve tests, which presumably reflects Executive Functions (EF) (convergent validity), while the scales of difficulty in emotional control and in social behavior are not related to these cognitive skills (discriminant validity).

Despite the problems related to the use of self-reported measures for assessing subjective cognitive decline in the elderly with and without associated pathology, the PSI may represent an easy-to-apply objective instrument that can assist in clinical practice in identifying behavioral symptoms derived from prefrontal deficits. According to Rabin et al. (13), the measures used are not standardized and most assess memory-related complaints without correlations with biomarkers.

The present study had the following objectives: to adapt the PSI (abbreviated version) to Brazilian Portuguese; evaluate its psychometric properties in an elderly population; investigate the evidence of convergent validity of the PSI with the Geriatric Depression Scale (GDS)-15, the Mini-Mental State Examination (MMSE), and the FAB; and verify whether the PSI could distinguish between groups with and without the diagnosis of probable AD.

## MATERIAL AND METHODS

### Participants

Elderly individuals (>60 years) make up 10.32% of the population of João Pessoa (PB, located in the state of Paraíba) (14). The Portney and Atkins equation (15) was adopted implementing a 95% confidence interval and a 5% margin of error to estimate a representative sample of elderly individuals: this calculation identified that 142 people would be required. Thus, we recruited 206 elderly, including both sexes, over the age of 60 years, and with at least 1 year of formal education, from social centers, residents' associations, and institutions for the elderly in the PB metropolitan area. A subjective clinical evaluation was performed collecting data on signs and symptoms, as well as the application of cognitive screening instruments such as MMSE and Cognitive Assessment test of Montreal (MOCA). Participants who had psychiatric disorders, severe motor and/or cognitive disorders, substance abuse, and those who dropped out at any stage of the study were excluded.

Additionally, 50 elderly of both sexes, aged over 60 years and with at least 1 year of formal education, recruited from the cities of PB and Recife (PE, Paraiba and Pernambuco), constituted the clinical group with mild/moderate AD. A broad clinical interview was conducted with the caregivers to screen the probable AD level. Additionally, the Clinical Dementia Rating (CDR), considering the criteria of the Diagnostic and Statistical Manual of Mental Disorders-5 (16) and the National Institute of Neurological and Communicative Disorders and Stroke (NINCDS) and Alzheimer’s Disease and Related Disorders Association (NINCDS-ADRDA) (17), was applied, and only individuals of CDR 1 and 2 were included, while those with CDR 3 (probable end-stage AD) were excluded. Those who reported vascular lesions, had a diagnosis of psychiatric disorders or psychoactive substance dependence, or severe visual and/or hearing impairment that could impair the performance evaluation, and those who refused to complete any cognitive test were excluded.

### Instruments

#### Prefrontal Symptoms Inventory

The PSI, an instrument of Spanish origin (10), was originally composed of 46 items (α>0.94), with psychometric properties, but also includes an abbreviated version containing 20 items. The latter was used in the present study (α>0.89). It is answered on a Likert scale (0-4 points). The objective of the PSI is to explore behaviors in everyday life that are related to changes in the prefrontal cortex, in the behavioral, emotional, and social domains. It consists of three theoretically independent subscales. The first subscale (composed of 12 items) assesses behavioral problems, including motivational problems, executive control, and attention problems; the second (composed of four items) assesses problems in social behavior; and the third (composed of four items) evaluates difficulties in emotional control.

### FAB

The FAB has been validated for Brazilian Portuguese by Beato et al. (18). It consists of six subtests: abstract reasoning, mental flexibility, cognitive programming for motor action, sensitivity to interference, inhibitory control, and autonomy in the internal control of environmental stimuli. Each test corresponds to an activity controlled by the frontal lobe, which allows the FAB to detect executive dysfunction, which refers to a deficit in brain functions essential for directed, flexible, and adaptive behavior, particularly in new situations. Each test is rated between 0 and 3, with the total corresponding to the sum of the scores of each activity (score range=0-18 points, with 18 indicating the best performance).

### MMSE

The MMSE was evaluates five dimensions (orientation, attention, concentration, memory, calculus, language, and praxis) and was based on theoretical analysis and clinical practice. It is the test most commonly used to assess cognitive function because can be rapidly (around 10 minutes) and easily administered, and requires no specific materials. It is used as a screening tool, and does not replace a more detailed evaluation, because even though it evaluates several domains, indicating the functions that require investigation, it cannot serve as a diagnostic test. The adaptation developed by Brucki et al. (19), with a maximum score of 30 points, was used in the present study.

### GDS

The GDS is a dichotomous self-reported instrument (based on yes or no responses) commonly used for clinically assessing depression in the elderly. Version 15 of the GDS (GDS-15) was used in the present study (20). The adopted cut-off (≥6) yields a sensitivity of 85.4% and a specificity of 73.9% for screening for depressive symptoms. The internal consistency of the scale has a Cronbach’s alpha of 0.81.

### Procedures

The research was approved by the research ethics committee of the Onofre Lopes University Hospital of the Federal University of Rio Grande do Norte (CAEE 50929115.7.0000.5292).

The adaptation of the PSI (20 items) for the Brazilian population followed the guidelines of Borsa et al. (21). It was independently translated into Portuguese by two bilingual (Spanish-Portuguese) experts; then, the instrument was sent to three expert evaluators in psychological assessment, neuropsychology, and gerontology. The items were judged in terms of adequacy, clarity, and comprehension (1, adequate; 2, partially adequate; 3, inadequate).

Items on which experts agreed at a rate of at least 90% were included in the Brazilian version of the scale, without any revision. Items on which experts agreed at a rate of 70-80% were reviewed according to suggestions. In the next step, the PSI was individually applied as a clinical interview to a group of 10 elderly of both sexes. They were asked to identify items that they did not understand or presented difficulties in answering. Some items were reviewed considering the feedback received from the group, to ensure the compatibility of the items with Brazilian culture.

A backward translation was performed by two other bilingual professionals (Spanish-Portuguese) to test the conceptual equivalence of the instrument. The retranslation did not remain the same as the original instrument in linguistic terms, but the items reflected the same content. Therefore, the Portuguese-adapted instrument comprised the 20 items included in the original version.

All participants provided written informed consent prior to any procedure in the study. Each volunteer underwent an individual session of approximately 30 minutes with their respective investigators (who were psychologists). This included a structured interview to collect demographic, physical, and mental data, as well as their responses to the MMSE and GDS, which were used for clinical assessment of the participants (mental state and depressive symptoms). Then, the volunteers were assessed based on the FAB and PSI. Participants with probable AD were interviewed together with their respective caregiver to minimize anosognosia (22).

### Data analysis

Data were grouped and analyzed using Statistical Package for the Social Sciences (SPSS) version 22 software. Descriptive and inference statistics (Student’s *t*-test) were used to analyze the demographic and clinical characteristics of the clinical and non-clinical group. The factor structure was tested using confirmatory factor analysis (CFA) using R Studio software and the Lavaan and SemPlot packages with the Weighted Least Squares Mean-Variance Adjusted estimator. This analysis also considered the following adjustment indices to measure the quality of the model. A chi-square value with degrees of freedom (χ^2^/df) <5; a Comparative Fit Index >0.90, indicating a good fit; Root Mean Square Error of Approximation <0.05, but accepted up to 0.08; Standardized Root Mean Square Residual (SRMR) <0.10, to assess discrepancies between the observed and the expected model; and a Goodness of Fit Index >0.90. The expected cross validation index proposes the expected cross validation index that measures how well a model can predict future sample covariances, with the smallest value considered to be the most acceptable (23). Furthermore, McDonald’s Ω and Cronbach’s α were presented for reliability analysis. Pearson’s correlation coefficient was used to verify the convergent validity of the instrument, and differences between groups were tested using Student’s *t*-test (effect sizes estimated using Cohen’s d).

## RESULTS

The clinical and sociodemographic data of the sample are presented in Table 1. The group with probable AD had an average age of 74.5 years (minimum=60 years; maximum=93 years, standard deviation [SD]=9.72 years); the majority were female (70%), more than half had more than 8 years of education (52%), almost two-thirds were married (60%), and more than half were without frequent engagement in physical activities (52%). The non-clinical group had an average age of 74.0 years (minimum=60 years, maximum=100 years, SD=8.52); most were also women (72.8%), half had more than 8 years of education (50%), about one-third were widowed (32.8%), more than half had no subjective complaints of cognitive problems (59.7%), and more than half regularly engaged in physical activity (56.3%).

The clinical group (probable AD) and the group without pathology (non-clinical group) showed statistically significant differences in marital status (χ^2^ (3)=28.23, *p*<0.001) and subjective complaints of cognitive decline (χ^2^ (1)=57.46, *p*<0.001). The clinical group had a statistically significantly lower mean MMSE score than the non-clinical group (t (157)=6.29; *p*<0.001; d=0.69; 95%CI [3.45; 6.61]). The mean score on the GDS was below six points in both groups (cut-off point for depression symptoms), and the clinical group showed more depressive symptoms than the non-clinical group (t (157)=-5.60; *p*<0.001; d=0.69, 95% CI [-7.91; -348]).

### PSI CFA

Uni- and tri-factorial models were examined using CFA to test the factorial structure of the PSI. It was verified that both models presented good adjustment indices (Table 2). However, the originally proposed tri-factorial model presented better adequacy adjustments, since a smaller SRMR value indicates les marked discrepancies between the expected and observed value of the model.

However, McDonald’s Ω was not satisfactory for the tri-factorial model, because it did not reach the acceptable minimum of 0.60. Only the behavioral problems factor (Ω=0.82) was adequate, but those for social conduct (Ω=0.50) and emotional control (Ω=0.51) were inadequate. Thus, the PSI could not reliably measure what it intended to measure. The uni-factorial model presented an Ω of 0.83, indicating that this is the best model for use in an older adult population with and without pathology.

**Figure 1:**
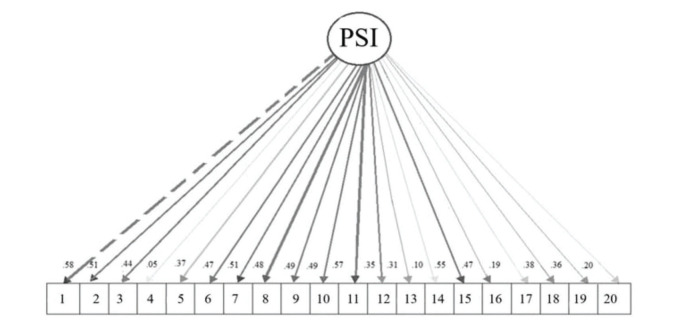
PSI factorial structure. The circle represents the construct (latent variable) and the squares constitute the observable variables (instrument items); the denser the lines, the more representative the item (higher factor load) (24). PSI, Prefrontal Systems Investory.

We observed the loading weights for the general latent trait to confirm this structure as the most appropriate, as shown in Figure 1, graphically represented by the semPlot package (24). Items 4, 14, 17, and 20 did not present satisfactory loads (0.05, 0.10, 0.19, and 0.19, respectively), indicating that they do not belong to a single factor. Thus, we chose to exclude them: item 4 (“I cry or laugh easily”) of the emotional control factor and three items of social conduct: item 14 (“I tell inappropriate jokes in inappropriate situations), item 17 (“I comment on the intimate matters of others”), and item 20 (“I make inappropriate sexual comments in any environment”). The remaining 16 items were related to behavioral problems (attention, motivation, executive, and emotional control) and all had standardized factor loads exceeding 0.35 (Table 3). In this sense, the best model is that which minimized the smallest chance of error with items that actually represent the latent trait; thus, the best model was the uni-factorial model. The internal coefficient was also statistically satisfactory (α=0.80).

### Convergent validity

As shown in Table 4, the non-clinical group showed statistically significantly weak and moderate correlations of the PSI-16 with the GDS-15, MMSE, FAB, and their six subtests, indicating that there was convergence between the items assessed by the PSI-16 and the other instruments that measure aspects related to prefrontal cortex function and emotional problems. However, in the clinical group, there were only moderately significantly negative correlations of the PSI-16 with the MMSE and total FAB score, and the conceptualization subtest score.

### Differences between groups and effect of age and education on PSI-16 performance

There were statistically significant differences between the non-clinical and clinical groups regarding PSI-16 performance (t (254)=-4.47, *p*<0.001; d=0.65; 95% CI [-9.81; -3.81]), in which the clinical group achieved higher scores (M=25.78; SD=11.4) than the non-clinical and clinical groups (M=18.97; SD=9.18). There were also correlations between age, education, and group performance on the PSI. In the non-clinical group, there was a significant positive although weak correlation between age and PSI-16 (r=0.186, *p*<0.05), and a negative (moderate) correlation between education and PSI-16 (r=-0.357, *p*<0.001), demonstrating that the lower the age and higher the educational level, the lower the prefrontal symptoms. There were no statistically significant correlations in the clinical group.

## DISCUSSION

The present study sought to adapt the shortened version of the PSI-20 to Brazilian Portuguese and find evidence of its validity in a population of elderly individuals with and without neurological pathology. We aimed to gather evidence pertaining to the adequacy of this instrument for detecting prefrontal problems, which may serve health professionals in clinical practice. No previous study had reported cultural and linguistic adaptations of the instrument in Portuguese, which makes it impossible to compare results, and no previous study had reported its application in a specific population of elderly individuals.

The norms for scale validation were obeyed in this study (25), and the theoretical conformity with the original instrument was proven. The tri-factorial model proposed in the original study by Ruiz-Sánchez de León et al. (10) and a uni-factorial model both presented satisfactory adjustment indices in this study. However, only the single-factor model showed accurate internal consistency, given that two of the factors (*i.e.*, emotional and social) did not have adequate internal coefficients (26), even when using McDonald’s Ω, which is based on the proportion of the common variance in a test, and thus performs better than other indices, such as Cronbach’s α (27).

Ruiz-Sánchez de León et al. (12) initially grouped the main factors of the PSI into three closely related components: motivation (impulse and interest in initiating a behavior); execution control (ability to develop a plan, have cognitive flexibility, and problem-solving); and attention (attention management) (1). However, the model proposed in the present study indicates that the emotional aspects must also be considered within this structural set, meaning that the items were each involved in generating a single factor.

Individuals do not use one function independently of others, and thus it was plausible that there would not be full linearity in the responses. From the neuroanatomic point of view, the PFC region is an area of interconnections. The PFC integrates primary sensorimotor processes and modulates higher order cognitive functions. Processes that require planning, attention, working memory, problem-solving, and cognitive flexibility require the integrity of the dorsolateral PFC (DLPFC). Mental elaboration through response selection, conflict resolution, selective attention, and self-monitoring requires the integrity of the superior medial PFC (28).

Mental elaborations through social adjustment and response inhibition require the integrity of the ventral medial PFC (ventral MPFC) and the orbitofrontal cortex (OFC). The dorsal anterior cingulate cortex (dACC) is involved in detecting and monitoring affective and non-affective stimuli, and the ventral/rostral ACC primarily works with the posterior parietal and DLPFC regions to regulate affective responses (29).

Moreover, convergent validity was assessed using the MMSE, FAB, and GDS-15 in the non-clinical group. These instruments are used for cognitive screening (MMSE) and measuring aspects related to executive dysfunction (FAB). We found that the PSI-16 was negatively correlated (mild and moderate correlations) with the MMSE, FAB, and GDS-15, which indicated that the higher the prefrontal symptoms, the worse the cognitive performance and depressive symptoms. The PSI-16 behavioral items assess daily difficulties related to cognitive performance deficits, confirming the findings of Pedrero-Pérez et al. (12).

The ventral MPFC and DLPFC (MPFC) are implicated in the emotional regulation process and are structurally connected with several key areas, such as the posterior cingulate cortex and hippocampus, as well as with the caudate amygdala, insula, putamen (the reward system), middle temporal cortex (key for Theory of Mind), dorsal anterior cingulate cortex, and ventrolateral PFC (involved in inhibitory control and attention) (30). Therefore, mood changes caused by depressive symptoms may interact with the emotional, cognitive, and behavioral regulation as assessed by the PSI-16 (31).

However, in the clinical group, the PSI-16 only correlated significantly (moderately) with instruments that measured cognition. A longitudinal study developed by Péràs et al. (32) demonstrated that there was a noticeable decline in activities of daily living 2 years before the diagnosis of AD, and these changes occurred concurrently with executive decline. This was in line with our findings, where the clinical group exhibited convergence between the cognitive alterations noted on the instruments, demonstrating that difficulty in conceptualizing (FAB) would be more strongly related to performance on the PSI-16.

In the comparison of the groups, it was clear that participants with a diagnosis of probable AD exhibited more prefrontal symptoms. The study by Ruiz-Sánchez de León et al. (12) reported average normative values of 22.28 points (SD: 12.78) for a clinical population with degenerative diseases and of 26.9 points (SD: 12.8) for a population with addiction behavior in chemical substances, compared to a normative value of 12.2 (SD: 10) for a non-clinical population. The mean scores in the present study were higher, despite our scale including four fewer items.

Of course, a certain amount of cognitive decline is expected in the aging process; this may vary considerably between individuals and cognitive domains, with some functions appearing more susceptible than others to the effects of aging. Declines in cognition tend to be more pronounced in EF, particularly in individuals with some form of dementia (33).

One of the models of EF advocated by Zelazo et al. (34) emphasizes both cognitive and socio-affective aspects, classifying them into “cold” and “hot” executive processes. The “cold” components involve the most logical and cognitive aspects, such as logical and abstract thinking, planning, problem-solving, and working memory. On the other hand, “hot” processes are more related to emotional aspects, such as the regulation of affection, motivation, social behavior, decision-making, reward experience, personal interpretations, and moral judgment.

Importantly, in the non-clinical group, the PSI-16 performance was found to correlate significantly with age and education. It is well established in the literature that age may be a risk factor for cognitive decline and emotional conflict. In addition, the higher the educational level, the greater the intellectual stimulation and promotion of axonal and synaptic growth, enabling the formation of a cognitive reserve that protects the brain from pathological processes (35).

In the clinical group, no significant correlations of PSI-16 performance with age and education were noted. Some studies have reported that one of the characteristics of AD is anosognosia, which emphasizes a lack of awareness about deficits associated with the disease. This is a common feature and has implications for the ability to manage risk, and can also complicate research using self-reports. Thus, we sought to minimize this effect by confirming the responses of participants with caregivers, but these results could be more accurate if there were comparisons with the information provided by professionals, such as in the study of Huertas-Hoyas et al. (36). In addition, longitudinal follow-up of the patients is necessary.

This study contribute to understanding the psychometric properties of the PSI-16 in an elderly population, and highlights the need for further studies of this nature in Brazil, to investigate cognitive, behavioral, and emotional symptoms from a clinical point of view. The PSI-16 used here provided a quantitative and qualitative assessment of symptomatology, presenting some advantages from the clinical point of view, in addition to being effective and economically efficient. However, the study was not exempt from limitations, such as the small sample size of both the clinical and non-clinical groups, making more robust statistical analyses unfeasible. There were also a substantial number of participants with a low education level, which may also have impaired understanding of the PSI-16 items, while the age of the population may have imposed both pathological and non-pathological difficulties.

## CONCLUSIONS

The present study evaluated the psychometric properties of an abbreviated, Portuguese-adapted version of the PSI for use in the elderly population with and without neurological pathology. In this study, the tri-factorial model, proposed in the original study by Ruiz-Sánchez de León, et al. (10), and a uni-factorial model, was tested; however, the latter demonstrated more accurate internal consistency, and included 16 items. Our results imply that when assessing prefrontal symptoms, the interaction between behavioral, cognitive, and emotional aspects should be considered. For the non-clinical group, we found that the more severe the prefrontal symptoms, the worse the cognitive performance and depressive symptoms, whereas in individuals with probable AD, the prefrontal symptoms were related only to the cognitive elements, since they are more latent, in view of the pathology. The PSI-16 scores for prefrontal symptoms were higher in the clinical than in the non-clinical group. In general, the PSI-16 is a valid and reliable tool for clinical assessment of elderly individuals with and without neurodegenerative pathology.

## AUTHOR CONTRIBUTIONS

Ferreira OD and Alchieri JC conceived and designed the study, were involved in the analysis and interpretation of the data, and contributed to revising the draft. Barbosa LN and Ferreira OD participated in the recruitment and collection of elderly participants.

## Figures and Tables

**Table 1:** Sociodemographic and clinical data of elderly with and without AD.

		Total (n=256)	Non-clinical sample (n=206)	Clinical sample (n=50)
Age, years; Mean (SD)		74.10 (8.75)	74.0 (8.52)	74.5 (9.72)
Sex	Female	185 (72%)	150 (72.8%)	35 (70%)
	Male	71 (28%)	56 (27.2%)	15 (30%)
Education level	Low (under 4 years)	97 (37.9%)	83 (40.3%)	14 (28%)
	Middle (between 4 and 8 years)	30 (11.7%)	20 (9.7%)	10 (20%)
	High (above 8 years)	129 (50.4%)	103 (50%)	26 (52%)
Civil status	Single	51 (19.9%)	48 (23.3%)	3 (6%)
	Married	80 (31.3%)	50 (24.3%)	30 (60%)
	Divorced	41 (16%%)	39 (18.9)	2 (4%)
	Widowed	84 (32.8%)	59 (33.5%)	15 (30%)
Medication	Yes	239 (93.4%)	189 (91.7%)	50 (100%)
	No	17 (6.6%)	17 (8.3%)	0
Subjective complaints in cognition	Yes	133 (52%)	83 (40.3%)	50 (100%)
	No	123 (48%)	123 (59.7%)	0
Physical activity	Yes	140 (54.7%)	116 (56.3)	24 (48%)
	No	116 (45.3%)	90 (43.7%)	26 (52%)
MMSE, Mean (SD)		22.84 (6.79)	25.38 (4.37)	20.34 (5.30)
GDS, Mean (SD)		3.79 (3.01)	2.96 (2.34)	4.52 (3.61)

Note: AD, Alzheimer’s disease; MMSE, Mini-Mental State Examination; GDS, Geriatric Depression Scale; SD, Standard deviation.

**Table 2:** Comparison of PSI factor structure models.

Models	χ^2^	Df	χ^2^/df	CFI	GFI	TLI	RMSEA (95%CI)	SRMR	ECVI	Δχ^2^(df)
Uni	222.16	170	4.35	0.96	0.91	0.95	0.03 (0.03-0.06)	0.07	1.18	-
Tri	146.96	149	2.65	1.00	0.97	1.00	0.00 (0.00-0.02)	0.05	0.98	75.2 (21)**

Note: χ2, chi-square test; DF, Degrees of Freedom; χ2/df, comparison between models; CFI, Comparative Fit Index; CI, Confidence Interval; GFI, Goodness of Fit Index; TLI, Tucker-Lewis index; RMSEA, Root Mean Square Error of Approximation; SRMR, Standardized Root Mean Square Residual; ECVI, Expected Cross Validation Index; **p<0.001.

**Table 3:** Factorial loads for Prefrontal Systems Inventory items (n=256).

Items	Factor Loads
1. I have difficulty starting an activity due to lack of initiative	0.59
2. It is very hard to focus on something	0.53
3. I cannot do two things at once, such as tidying up and talking on the phone	0.50
4. I get bored with anything and easily get annoyed	0.44
5. I have trouble changing the subject during conversations	0.53
6. I get slow as if I'm almost asleep.	0.52
7. I find it difficult to make decisions	0.52
8. I forget the things I have to do until someone reminds me	0.52
9. I only do what I have to do when someone tells me	0.58
10. I have trouble keeping up with a movie or book	0.60
11. It is hard to think of things in advance or plan for the future	0.37
12. My emotions can change from happiness to sadness easily	0.36
13. It is hard to do things out of disposition	0.58
14. It is hard to plan things in advance	0.50
15. I do or say embarrassing things	0.51
16. I explode emotionally for no apparent reason	0.45

Note: All factor loadings are statistically significant.

**Table 4:** Correlation of the PSI-16 with the GDS-15, MMSE, and FAB in the Non-Clinical and Clinical Groups.

	Non-clinical group (n=206)	Clinical group (n=50)
Variables	PSI-16	PSI-16
GDS-15	0.534^**^	---
MMSE	-0.240^**^	-0.251*
FAB	-0.403^**^	-0.296*
Conceptualization	-0.164*	-0.346*
Mental flexibility	-0.374^**^	---
Programming	-0.190^**^	---
Interference	-0.358^**^	---
Inhibitory control	-0.356^**^	---
Environmental autonomy	---	---

Note: GDS-15, Geriatric Depression Scale version 15; MMSE, Mini-Mental State Examination; FAB, Frontal Assessment Battery; PSI-16, Prefrontal Systems Investory-16.

*p<0.05; **p<0.01.
